# CCR6 controls autoimmune but not innate immunity‐driven experimental arthritis

**DOI:** 10.1111/jcmm.13783

**Published:** 2018-08-22

**Authors:** Michael Bonelli, Antonia Puchner, Lisa Göschl, Silvia Hayer, Birgit Niederreiter, Guenter Steiner, Katharina Tillmann, Roberto Plasenzotti, Bruno Podesser, Philippe Georgel, Josef Smolen, Clemens Scheinecker, Stephan Blüml

**Affiliations:** ^1^ Internal Medicine III Division of Rheumatology Medical University of Vienna Vienna Austria; ^2^ Division of Biomedical Research Medical University of Vienna Vienna Austria; ^3^ NSERM UMR_S 1109 Université de Strasbourg France

**Keywords:** adaptive immune system, arthritis, CCR6, chemokine receptors, CIA, innate immune system, rheumatoid arthritis

## Abstract

Rheumatoid arthritis (RA) is a chronic inflammatory autoimmune disease, characterized by synovial infiltration of various inflammatory cells. Chemokines are involved in controlling the recruitment of different cell types into the synovial membrane. The role of CCR6 in the development of arthritis so far remains unclear. In this study, we investigated the role of CCR6 in the pathogenesis of arthritis using three different murine arthritis models. Compared to WT animals, *CCR6* ^*−/−*^ mice developed less clinical signs of arthritis in the collagen‐induced arthritis model but not in the K/BxN serum transfer arthritis model and in the human tumour necrosis factor transgenic arthritis model, suggesting a defect in adaptive effector functions but intact innate effector functions in the development of arthritis in *CCR6* ^*−/−*^ animals. In line with this, anti‐collagen antibody levels were significantly reduced in *CCR6* ^*−/−*^ mice compared with *WT* mice. Moreover, we demonstrate enhanced osteoclastogenesis in vitro in *CCR6* ^*−/−*^ mice compared with *WT* mice. However, we did not detect differences in bone mass under steady state conditions in vivo between *WT* and CCR6‐deficient mice. These data suggest that CCR6 is crucially involved in adaptive but not in innate immunity‐driven arthritis. CCR6 or its chemokine ligand CCL20 might represent a possible new target for the treatment of RA.

**Key messages**

- CCR6 affects the development of CIA
- CCR6 is not involved in the TNF transgenic or serum transfer arthritis model
- CCR6 is involved in adaptive but not in innate immunity‐driven arthritis.

## 1 INTRODUCTION

Rheumatoid arthritis (RA) is a chronic autoimmune disease characterized by joint inflammation and tissue destruction. The inflammatory reaction in the synovium is characterized by the activation and proliferation of fibroblast‐like synoviocytes as well as the recruitment of leucocytes such as neutrophil granulocytes, monocytes/macrophages, dendritic cells and lymphocytes such as T and B cells. All of these cell types have been reported to contribute to the inflammatory process in RA patients.

Migration of inflammatory cells is mediated by a set of receptors which respond to environmental cues and direct the migration of inflammatory cells to the site of inflammation. Among them, the chemokine receptor 6 (CCR6) has been demonstrated to be expressed on a number of cell types important in the pathogenesis of inflammatory arthritis. CCR6 has been shown to characterize a subset of CD4^+^ T cells, the IL‐17‐producing Th17 cells.^1^, ^2^ CCR6^+^ T cells have been detected in the inflamed synovium and the peripheral blood of RA patients.^3^, ^4^, ^5^, ^6^ CCR6^+^ effector T cells can produce various cytokines like IL‐17A, IL‐17F, IL‐22, IL‐26, TNF‐α, IFN or GM‐CSF and therefore contribute to the development of RA by activation of monocytes, neutrophils, synovial fibroblasts and osteoclast differentiation.^7^, ^8^ The ligand for CCR6, CCL20, is highly expressed at sites of inflammation, including the inflamed synovium of RA patients and therefore promotes the recruitment of CCR6‐positive cells.^9^, ^10^, ^11^ In T cell‐driven experimental arthritis models as well as in patients with RA, the CCR6/CCL20 axis has convincingly been shown to promote the recruitment of Th17 cells into the inflamed synovial membrane.^11^

In addition to Th17 cells, CCR6 is also expressed on memory B cells and therefore might contribute to the migration of this cell population.^12^ CCR6 was also found on activated antigen‐specific B cells. Moreover, CCR6 has also been shown to be expressed on innate immune cells, including dendritic cells, granulocytes, macrophages and NK cells.^13^, ^14^, ^15^, ^16^, ^17^, ^18^ CCR6 was also expressed on infiltrating mononuclear cells in the synovium of RA patients.^19^

In patients with RA, CCR6 expression on monocytes has been shown to correlate with the potential of monocytes to differentiate into osteoclasts ex vivo, suggesting a possible role of CCR6 on monocytes in the recruitment of osteoclast precursors to the joint.^20^ However, despite the evidence for the importance of CCR6 in various cell types, which are known drivers for the development of RA, the role of CCR6 in the development of arthritis beyond T cell‐dependent mechanisms has not been addressed. In this study, we therefore studied the effect of CCR6 deficiency in three different models of experimental arthritis, each with distinct, non‐overlapping pathomechanisms.

## 2 METHODS

### 2.1 Mice

C57BL/6 mice and *CCR6* ^*−/−*^ mice were obtained from The Jackson Laboratory. All experiments were approved by the local ethics committee.

### 2.2 TNF transgenic mouse model

CCR6^−/−^ mice were crossed into human TNF‐α transgenic mice (Tg197 strain, C57BL/6 genetic background; originally generated by the group of George Kollias (Fleming Institute, Athens, Greece)^21^) to obtain hTNFtg/CCR6^−/−^ mice. Mice were maintained under conventional housing conditions (humidity 50%, 22°C, 12‐hour light/12‐hour dark cycle). All experiments were performed in females. Age‐matched non‐transgenic female littermates were used as controls. All experiments were approved by the local ethical committee, Federal Ministry of Science, Research and Economics.

### 2.3 Induction of CIA

C57BL/6 mice were immunized subcutaneously with 50 μg chicken type II collagen (Sigma‐Aldrich, Vienna, Austria) in 50 μL H2O, emulsified in 50 μL Freund's complete adjuvant that was enriched with 10 μg/mL Mycobacterium tuberculosis (H37Ra; Difco/BD Biosciences, San Jose, CA, USA), on day 1 and day 21. Mice in this model are expected to develop arthritis between week 3 and week 10 and were evaluated weekly for symptoms of arthritis using a semiquantitative scoring system that includes the degree of joint swelling and grip strength. Briefly, joint swelling was examined using a clinical score ranging from 0 to 3 (0 no swelling, 1 mild swelling of the toes and ankle, 2 moderate swelling of the toes and ankle and 3 severe swelling of the toes and ankle). In addition, the grip strength of each paw was analysed using a wire, 3 mm in diameter, to determine grip strength scores ranging from 0 to 3 (0 normal grip strength, 1 mildly reduced grip strength, 2 moderately reduced grip strength and 3 severely reduced grip strength). Assessments were performed in a blinded fashion. Animals were killed between week 3 and week 10 after disease induction.

### 2.4 Measurement of serum anti‐CII antibody levels

On day 30 after the first immunization, approximately 50 μL of blood was collected from each animal by bleeding animals from the tail vein. Serum samples were prepared, and anti‐CII antibody levels were determined by ELISA. Briefly, ELISA plates (Nunc, Rochester, NY) were coated overnight at 4°C with 0.5 μg/mL chicken CII in PBS. After washing with PBS containing 0.05% Tween‐20 (Pierce, Rockford, IL), non‐specific binding was blocked with PBS 3% gelatine for 1 hour at room temperature. After washing three times, serum samples diluted 1/10000 were added and incubated for 1 hour at room temperature. After four washes, horseradish peroxidase‐conjugated goat anti‐mouse IgG, IgG1, IgG2a and IgG2c (Southern Biotech, Birmingham, AL) were added and incubated at room temperature for 1 hour, followed by five washes. Plates were developed using 3,3′,5,5′‐tetramethylbenzidine (TMB) (Biomedica, Vienna, Austria) as substrate. The OD was measured at 405 nm using a microplate reader (Titertek, Huntsville, AL). The quantity of specific antibody was measured for each animal, and data are expressed as mean relative units of activity based on a standard anti‐CII serum that was generated from pooled sera of arthritic mice. Antibody values >0.15 units/mL were considered as positive.

### 2.5 Induction of serum transfer arthritis

After intraperitoneal application of 150 μL of K/BxN serum on day 1 and day 3, mice were killed on day 12 to prepare tissue samples for histology.

### 2.6 Evaluation of inflammation and local bone erosions by histological examination

Hind paws were fixed in formalin overnight and then decalcified in EDTA until the bones were pliable. Serial paraffin sections (2 μm) were stained with haematoxylin and eosin (H&E) or stained for tartrate‐resistant acid phosphatase (TRAP) activity. TRAP staining was performed using a leucocyte acid phosphatase staining kit (Sigma). For exact quantification of the areas of inflammation, H&E‐stained sections were evaluated using an Axioskop 2 microscope (Carl Zeiss Micro‐Imaging, Oberkochen, Germany) and Osteomeasure Analysis System (OsteoMetrics), which allows absolute quantification of areas in histological sections. The sum of the areas of inflammation for each single mouse was calculated by evaluating all tarsal joints. The same haematoxylin and eosin ‐stained sections were analysed similarly for the quantification of erosions. In addition, the number of osteoclasts was counted in TRAP‐stained serial sections.

### 2.7 In vitro osteoclast assay

Bone marrow cells were isolated from *WT* and *CCR6* ^−/−^ mice and cultured for 3 days in 100 ng/mL macrophage colony‐stimulating factor (M‐CSF) to enrich for monocyte/macrophages and were then cultured in 10% FCS/Dulbecco's modified Eagle's medium supplemented with 30 ng/mL M‐CSF and 50 ng/mL RANKL (both from R&D Systems) for another 3‐4 days. Osteoclasts were detected by the presence of TRAP+ multinucleated cells (>3 nuclei).

### 2.8 Statistical analysis

The unpaired *t* test was used to test statistically significant differences. A *P*‐value less than or equal to 0.05 was considered significant. Data were analysed for Gaussian distribution.

## 3 RESULTS

### 3.1 Deletion of CCR6 reduces the development of arthritis in the collagen‐induced arthritis model

**Figure 1:**
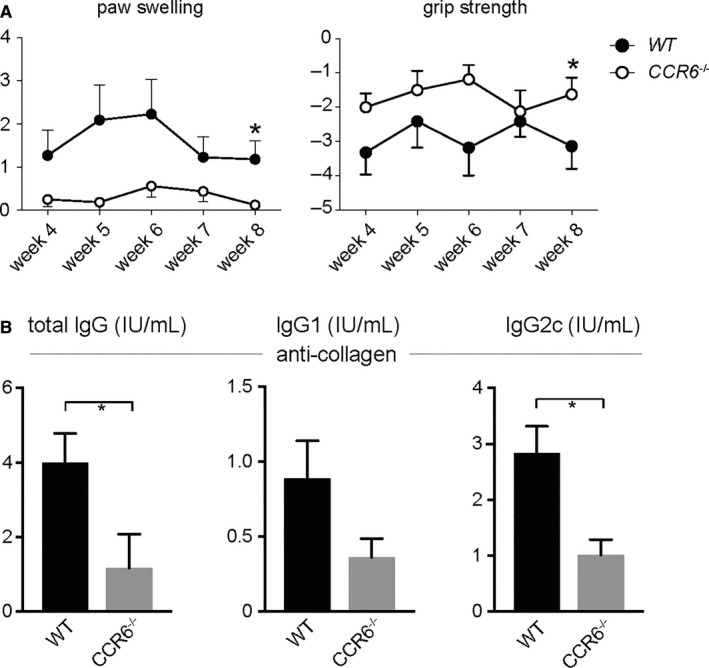
CCR6 Protects from the Development of CIA. CIA was induced in *WT* and *CCR6* ^−/−^ mice. A, Clinical quantification of paw swelling and grip strength in WT (n = 22) and CCR6^−/−^ (n = 16) mice after induction of CIA. Data are expressed as mean values ± standard error of the mean. A significant difference could be detected at week 8 for paw swelling and grip strength. B, Anti‐collagen antibodies from the serum of *WT* and *CCR6* ^−/−^ mice were measured by ELISA at week 4 after induction of CIA. Mean values ± standard error of the mean are shown. When indicated, statistical significance was shown as asterisks; *P‐value < 0.05

To address the role of CCR6 in arthritis, we first analysed *WT* and *CCR6* ^*−/−*^ mice in the collagen‐induced arthritis (CIA) model. As shown in Figure 1A, *CCR6* ^*−/−*^ mice developed significantly less severe arthritis. To elucidate the mechanisms of protection from CIA in *CCR6* ^*−/−*^ mice in further detail, we measured anti‐collagen antibody levels in the serum of *WT* and *CCR6* ^*−/−*^ mice. Of note, total IgG was significantly decreased in *CCR6* ^*−/−*^ mice as compared to *WT* mice. A more detailed analysis of IgG subclasses revealed a significant difference for IgG2c, which has been shown to be the pathogenic isotype in the CIA model (Figure 1B**)**. These data demonstrate the importance of CCR6 as part of the adaptive immune system for the development of arthritis

**Figure 2:**
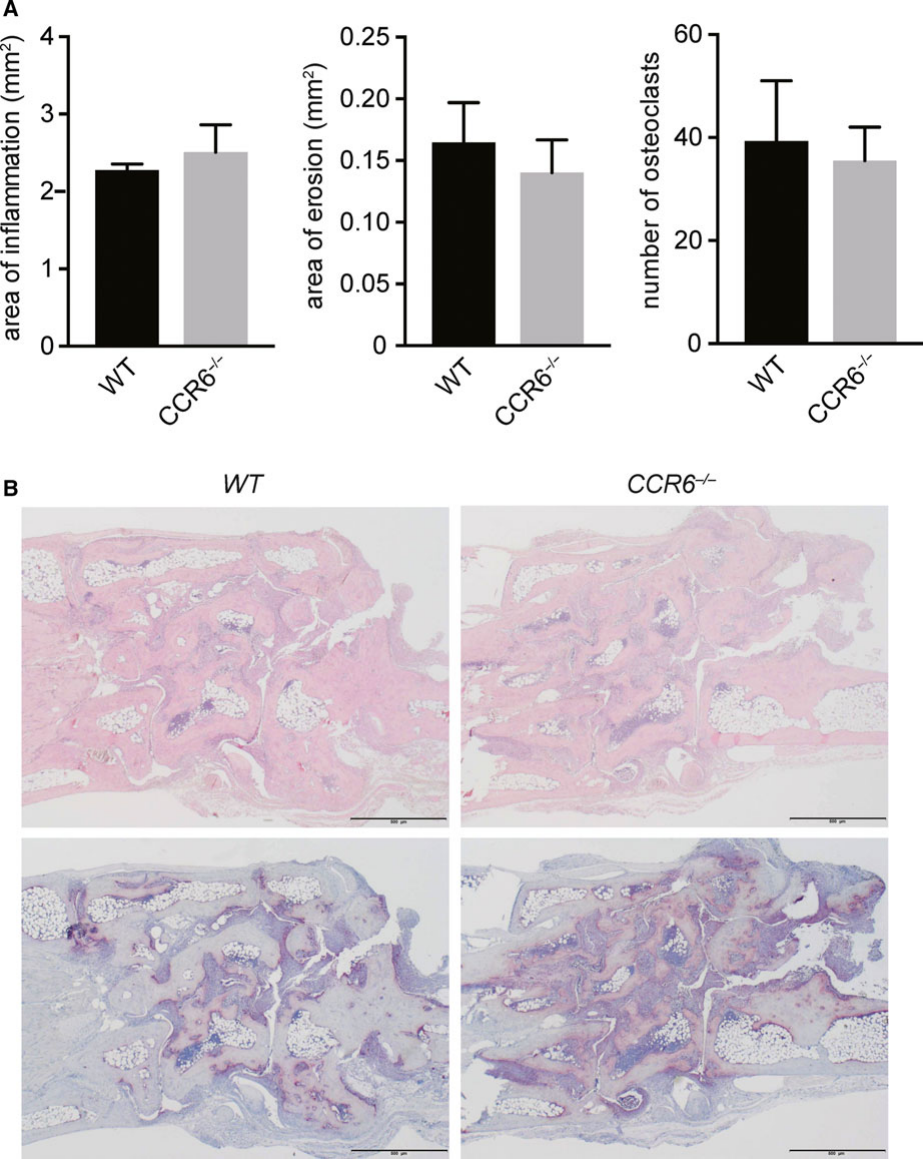
CCR6 does not Affect K/BxN Serum Transfer Arthritis. A, K/BxN serum transfer arthritis was induced in *WT* and *CCR6* ^−/−^ mice (n = 10). At day 12, mice were killed and analysed for histological signs of inflammation. Plots show quantitative histomorphometric analysis of hind paws of *WT* and *CCR6* ^−/−^ mice after induction of K/BxN serum transfer arthritis. Data are expressed as mean values ± standard error of the mean. *P ≤ 0.05, **P ≤ 0.01. B, Representative TRAP and haematoxylin and eosinstainings from histological sections are shown from *WT* and *CCR6* ^−/−^ *mice*. Mean values ± standard error of the mean are shown. When indicated, statistical significance was shown as asterisks; *P‐value < 0.05

To see whether CCR6 might be involved in the antibody‐mediated effector phase, we tested the effects of CCR6 deletion in a T and B cell inflammatory disease model. We therefore induced the K/BxN serum transfer arthritis in *WT* and *CCR6* ^*−/−*^ mice. All mice developed clinical signs of arthritis (data not shown). No difference could be detected between *WT* and *CCR6* ^*−/−*^ mice when we analysed the mice for histological signs of inflammation, erosions or the number of osteoclasts **(**Figure 2 **)**. These data suggest that CCR6 expression is important for the development of arthritis in CIA, but not for antibody‐mediated effector functions.

### 3.2 Increased osteoclastogenesis but regular bone density in *CCR6* ^−/−^ mice

**Figure 3:**
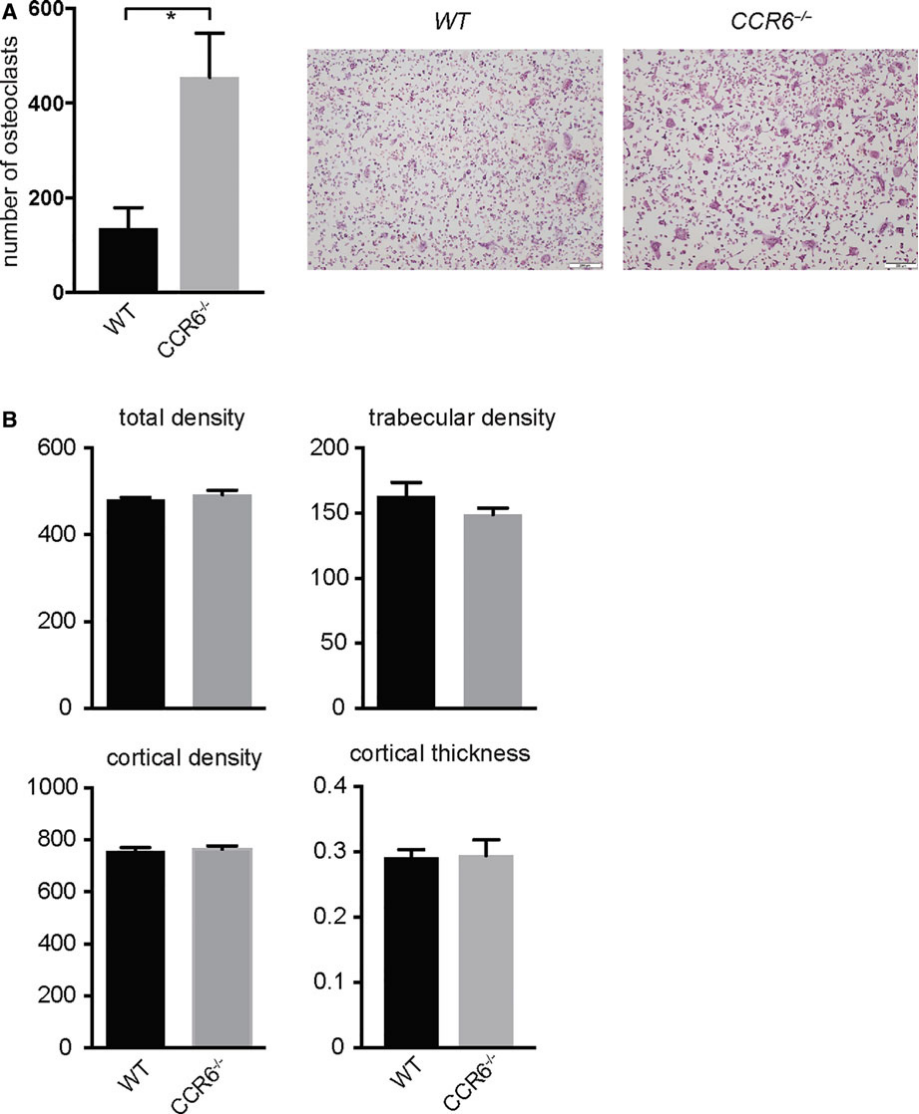
Regular Bone Density in *CCR6* ^−/−^ Mice. A, ex vivo osteoclastogenesis using bone marrow cells from *WT* and *CCR6* ^−/−^ mice cells (n = 3). Mean values ± standard error of the mean of quantitative analysis of TRAP^+^ multinucleated cells are shown in the left panel. Representative TRAP stainings are shown on the right panels. B, Bone density was analysed in *WT* and *CCR6* ^−/−^ mice by DEXA (n = 10 each). When indicated, statistical significance was shown as asterisks; *P‐value < 0.05. Mean values ± standard error of the mean are shown. When indicated, statistical significance was shown as asterisks; *P‐value < 0.05

As CCR6‐expressing monocytes have been suggested as osteoclast precursors in patients with RA, we next tested the potential of bone marrow cells of *WT* and *CCR6* ^−/−^ mice to differentiate into osteoclasts in an in vitro osteoclast assay. Osteoclastogenesis was significantly enhanced in vitro in bone marrow cells derived from *CCR6* ^−/−^ mice, suggesting that indeed CCR6 might be involved in osteoclastogenesis in vitro (Figure 3A**)**. To test the relevance of this finding in vivo, we analysed the bone mineral density of *WT* and *CCR6* ^−/−^ mice. However, no difference could be detected for cortical density, trabecular density or trabecular thickness, suggesting that CCR6 is not involved in bone turnover under steady state conditions (Figure 3B**)**.

### 3.3 CCR6 does not affect the development of arthritis in the TNF arthritis model

**Figure 4:**
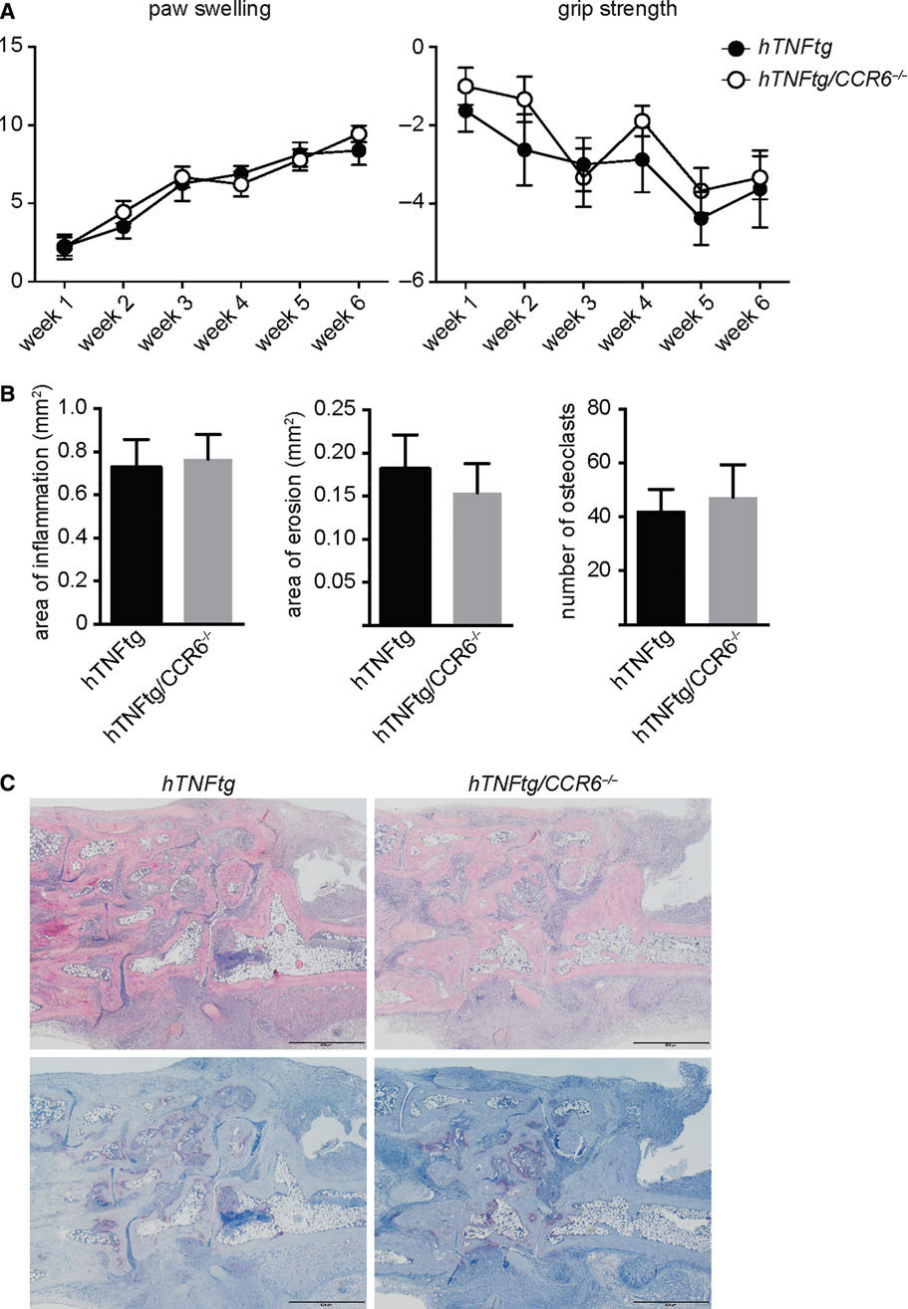
CCR6 does not Affect TNF‐Driven Chronic Arthritis. A, *CCR6* ^−/−^ mice were crossed to the human TNF‐α transgenic (hTNFtg) mice and scored for paw swelling and grip strength (n = 17). B, Quantitative histomorphometric analysis of inflammation in the synovial membrane of hTNFtg (n = 8) and hTNFtg/CCR6^−/−^ mice (n = 9) C, Representative histological sections (TRAP and haematoxylin and eosin stainings) are shown from *hTNFtg* and hTNFtg/*CCR6* ^−/−^ *mice*. Mean values ± Standard Error of the mean are shown. When indicated, statistical significance was shown as asterisks; *P‐value < 0.05

As CCR6 is expressed on many cell types that drive the inflammatory process in rheumatoid arthritis (RA), we also addressed the role of CCR6 in an additional arthritis model, the human TNF transgenic mouse model. To this end, *CCR6* ^−/−^ mice were crossed in the human TNF transgenic mice, which overexpress TNF and spontaneously develop arthritis. As shown in Figure 4, both *hTNFtg* and hTNFtg/*CCR6* ^−/−^ mice did develop clinical signs of arthritis. No difference between *hTNFtg* and *CCR6* ^−/−^/hTNFtg mice could be detected with respect to paw swelling and grip strength. In line with the clinical data, histological analysis of these mice revealed no difference in terms of histological signs of arthritis, such as area of erosion, area of inflammation or the number of osteoclasts between *hTNFtg* and *CCR6* ^−/−^/hTNFtg mice. These data show that under inflammatory conditions loss of CCR6 does not influence the development of synovitis both clinically or histologically. In particular, loss of CCR6 does not affect local generation of osteoclasts in this model of inflammatory arthritis. These data highlight the importance of CCR6 as part of the adaptive, but not of the innate immune system for the development of arthritis.

## 4 DISCUSSION

Previous studies have demonstrated CCR6 expression on a variety of cell types from the innate and adaptive immune system, which has been shown to be involved in the development of RA. The importance of CCR6 was further highlighted by GWAS studies, which have described a polymorphism of CCR6 in patients with RA.^22^, ^23^, ^24^, ^25^, ^26^ Moreover, an involvement of CCR6 in RA is further substantiated by the finding of elevated CCL20 levels in the joints of RA patients as compared to patients with osteoarthritis.^11^

On the other hand, whether CCR6 plays a role in the adaptive or the innate immune system for the development of arthritis has not been analysed so far. To decipher the exact role of CCR6, we therefore analysed *CCR6* ^*−/−*^ mice in three different arthritis models. This approach allowed us to study the role of CCR6 in spontaneous (hTNFtg) and induced (CIA and K/BxN serum transfer arthritis) models of arthritis. In addition, we could also dissect the role of CCR6 in innate immunity‐driven models of arthritis (hTNFtg and K/BxN serum transfer arthritis), as compared to the CIA model of arthritis that depends on the adaptive immune system.

To our surprise, deficiency of CCR6 did not affect the severity of arthritis in two different arthritis models, which are independent of T or B cells, namely the TNF transgenic arthritis and the serum transfer arthritis model. In sharp contrast, *CCR6* ^*−/−*^ mice developed a less severe arthritis in the CIA mouse model, which is dependent on the adaptive immune system. In particular, we detected reduced production of collagen‐specific antibodies in CCR6‐deficient mice compared to WT mice. This is in line with previous observations that deletion of CCR6 prevented memory B cells from the development of an effective secondary response upon antigen re/challenge, suggesting that CCR6 is not only essential for the migration but also for the ability to be recalled to their cognate antigen.^27^ These data further show that the CCR6/CCL20 axis does not play a significant role in innate effector mechanisms regulating pathology in inflammatory arthritis. In particular, our data disprove the notion that CCR6 is necessary for the migration of inflammatory cells into the arthritic joint. This is in contrast to other models of inflammatory diseases, where CCR6 has been shown to be important for the recruitment of inflammatory cells. An important role for CCR6 has been demonstrated for monocyte migration into atherosclerotic lesions and of immature DCs and monocytes into psoriatic skin lesions, suggesting an important role of the CCR6/CCL20 axis in the recruitment of pathogenic myelomonocytic cells in various inflammatory diseases.^28^, ^29^ In addition to cell trafficking, peritoneal macrophages isolated from *CCR6* ^*−/−*^ mice were found to express lower levels of inflammatory cytokines and nitric oxide following lipopolysaccharide stimulation compared to WT macrophages, suggesting that CCR6 might also be involved in the regulation of cytokine production in response to inflammatory stimuli.^30^

Furthermore, although we did detect an increased potential of bone marrow cells into osteoclasts in CCR6‐deficient animals in vitro, we did not find enhanced osteoclastogenesis in vivo in CCR6‐deficient mice, neither under steady state nor inflammatory conditions. A recent report speculates that CCR6 expression on CD14^+^ monocytes might be associated with enhanced osteoclastogenesis in RA patients. However, the authors did not compare the osteoclastogenic potential of CCR6^+^ and CCR6^−^ monocytes and hence could not show a direct link between expression of CCR6 on monocytes and osteoclastogenesis in vitro.^20^ Therefore, a direct comparison to our murine dataset is not possible.

Our findings are in line with previous reports which describe a role of CCR6 in the SKG arthritis model. Administration of anti‐CCR6 antibody suppressed the severity and onset of arthritis. This effect was found to be mediated by the binding of anti‐CCR6 antibody to the surface of B cells and a population of CD4^+^ T cells.^11^

More recent data also describe a pathogenic role CCR6^+^ T cells within the exFoxp3^+^ T cells, which have the potential to express RANKL and IL‐17 upon co‐culture with synovial fibroblasts and therefore enhance osteoclastogenesis.^31^ In addition, higher proportions of CCR6^+^ T cells within peripheral blood mononuclear cells were observed in RA patients with anti‐citrullinated antibodies (ACPAs). CCR6^+^ cells were inversely correlated with disease duration in ACPA‐RA patients.^32^ These data highlight the importance of CCR6 as part of the adaptive immune system to develop arthritis.

Further analysis of the mechanism that leads to a protection of the CIA in *CCR6* ^*−/−*^ mice revealed a difference in anti‐collagen antibodies in the serum of *CCR6* ^*−/−*^ mice as compared to *WT* mice. A detailed analysis of the IgG subclasses showed a difference in IgG2c antibody levels. These data are in line with a recent report, which describes CCR6 as an essential chemokine receptor for memory B cells for a recall response to their cognate antigen but not for their primary immune response.^27^

In conclusion, our data highlight for the first time the importance of CCR6 as part of the adaptive immune system for the development of arthritis, with no evidence of CCR6 being involved in innate immunity‐driven arthritis. These findings therefore identify the CCR6/CCL20 axis as a potential new target for the treatment of patients with seropositive RA.

## CONFLICT OF INTEREST

None declared.

## References

[1] Acosta‐Rodriguez EV , Rivino L , Geginat J , et al. Surface phenotype and antigenic specificity of human interleukin 17‐producing T helper memory cells. Nat Immunol. 2007;8:639‐646. 17486092 10.1038/ni1467

[2] Manel N , Unutmaz D , Littman DR . The differentiation of human T(H)‐17 cells requires transforming growth factor‐beta and induction of the nuclear receptor RORgammat. Nat Immunol. 2008;9:641‐649. 18454151 10.1038/ni.1610 PMC2597394

[3] van Hamburg JP , Asmawidjaja PS , Davelaar N , et al. Th17 cells, but not Th1 cells, from patients with early rheumatoid arthritis are potent inducers of matrix metalloproteinases and proinflammatory cytokines upon synovial fibroblast interaction, including autocrine interleukin‐17A production. Arthritis Rheum. 2010;63:73‐83. 10.1002/art.30093 20954258

[4] Leipe J , Grunke M , Dechant C , et al. Role of Th17 cells in human autoimmune arthritis. Arthritis Rheum. 2010;62:2876‐2885. 20583102 10.1002/art.27622

[5] Nistala K , Moncrieffe H , Newton KR , Varsani H , Hunter P , Wedderburn LR . Interleukin‐17‐producing T cells are enriched in the joints of children with arthritis, but have a reciprocal relationship to regulatory T cell numbers. Arthritis Rheum. 2008;58:875‐887. 18311821 10.1002/art.23291 PMC2675006

[6] Paulissen SMJ , van Hamburg JP , Davelaar N , Asmawidjaja PS , Hazes JM , Lubberts E . Synovial fibroblasts directly induce Th17 pathogenicity via the cyclooxygenase/prostaglandin E2 pathway, independent of IL‐23. J Immunol. 2013;191:1364‐1372. 23817417 10.4049/jimmunol.1300274

[7] Brennan FM , McInnes IB . Evidence that cytokines play a role in rheumatoid arthritis. J Clin Invest. 2008;118:3537‐3545. 18982160 10.1172/JCI36389 PMC2575731

[8] Chabaud M , Durand JM , Buchs N , et al. Human interleukin‐17: a T cell‐derived proinflammatory cytokine produced by the rheumatoid synovium. Arthritis Rheum. 1999;42:963‐970. 10323452 10.1002/1529-0131(199905)42:5<963::AID-ANR15>3.0.CO;2-E

[9] Baba M , Imai T , Nishimura M , et al. Identification of CCR6, the specific receptor for a novel lymphocyte‐directed CC chemokine LARC. J Biol Chem. 1997;272:14893‐14898. 9169459 10.1074/jbc.272.23.14893

[10] Liao F , Rabin RL , Smith CS , Sharma G , Nutman TB , Farber JM . CC‐chemokine receptor 6 is expressed on diverse memory subsets of T cells and determines responsiveness to macrophage inflammatory protein 3 alpha. J Immunol. 1999;162:186‐194. 9886385

[11] Hirota K , Yoshitomi H , Hashimoto M , et al. Preferential recruitment of CCR6‐expressing Th17 cells to inflamed joints via CCL20 in rheumatoid arthritis and its animal model. J Exp Med. 2007;204:2803‐2812. 18025126 10.1084/jem.20071397 PMC2118525

[12] Bhattacharya D , Cheah MT , Franco CB , et al. Transcriptional profiling of antigen‐dependent murine B cell differentiation and memory formation. J Immunol. 2007;179:6808‐6819. 17982071 10.4049/jimmunol.179.10.6808 PMC4517294

[13] Greaves DR , Wang W , Dairaghi DJ , et al. CCR6, a CC chemokine receptor that interacts with macrophage inflammatory protein 3alpha and is highly expressed in human dendritic cells. J Exp Med. 1997;186:837‐844. 9294138 10.1084/jem.186.6.837 PMC2199049

[14] Yang D , Howard OM , Chen Q , Oppenheim JJ . Cutting edge: immature dendritic cells generated from monocytes in the presence of TGF‐beta 1 express functional C‐C chemokine receptor 6. J Immunol. 1999;163:1737‐1741. 10438902

[15] Le Borgne M , Etchart N , Goubier A , et al. Dendritic cells rapidly recruited into epithelial tissues via CCR6/CCL20 are responsible for CD8 + T cell crosspriming in vivo. Immunity. 2006;24:191‐201. 16473831 10.1016/j.immuni.2006.01.005

[16] Salazar‐Gonzalez RM , Niess JH , Zammit DJ , et al. CCR6‐mediated dendritic cell activation of pathogen‐specific T cells in Peyer's patches. Immunity. 2006;24:623‐632. 16713979 10.1016/j.immuni.2006.02.015 PMC2855652

[17] Al‐Aoukaty A , Rolstad B , Giaid A , Maghazachi AA . MIP‐3alpha, MIP‐3beta and fractalkine induce the locomotion and the mobilization of intracellular calcium, and activate the heterotrimeric G proteins in human natural killer cells. Immunology. 1998;95:618‐624. 9893054 10.1046/j.1365-2567.1998.00603.x PMC1364361

[18] Cook DN , Prosser DM , Forster R , et al. CCR6 mediates dendritic cell localization, lymphocyte homeostasis, and immune responses in mucosal tissue. Immunity. 2000;12:495‐503. 10843382 10.1016/s1074-7613(00)80201-0

[19] Matsui T , Akahoshi T , Namai R , et al. Selective recruitment of CCR6‐expressing cells by increased production of MIP‐3α in rheumatoid arthritis. Clin Exp Immunol. 2001;125:155‐161. 11472439 10.1046/j.1365-2249.2001.01542.x PMC1906097

[20] Nanke Y , Kobashigawa T , Yago T , Kawamoto M , Yamanaka H , Kotake S . RANK Expression and osteoclastogenesis in human monocytes in peripheral blood from rheumatoid arthritis patients. Biomed Res Int. 2016;2016:4874195. 27822475 10.1155/2016/4874195 PMC5086380

[21] Keffer J , Probert L , Cazlaris H , et al. Transgenic mice expressing human tumour necrosis factor: a predictive genetic model of arthritis. EMBO J. 1991;10:4025‐4031. 1721867 10.1002/j.1460-2075.1991.tb04978.x PMC453150

[22] Hughes LB , Reynolds RJ , Brown EE , et al. Most common single‐nucleotide polymorphisms associated with rheumatoid arthritis in persons of European ancestry confer risk of rheumatoid arthritis in African Americans. Arthritis Rheum. 2010;62:3547‐3553. 21120996 10.1002/art.27732 PMC3030622

[23] Prasad P , Kumar A , Gupta R , Juyal RC , Thelma BK . Caucasian and Asian specific rheumatoid arthritis risk loci reveal limited replication and apparent allelic heterogeneity in north Indians. PLoS ONE. 2012;7:e31584. 22355377 10.1371/journal.pone.0031584 PMC3280307

[24] Kochi Y , Okada Y , Suzuki A , et al. A regulatory variant in CCR6 is associated with rheumatoid arthritis susceptibility. Nat Genet. 2010;42:515‐519. 20453841 10.1038/ng.583

[25] Stahl EA , Raychaudhuri S , Remmers EF , et al. Genome‐wide association study meta‐analysis identifies seven new rheumatoid arthritis risk loci. Nat Genet. 2010;42:508‐514. 20453842 10.1038/ng.582 PMC4243840

[26] Jiang L , Yin J , Ye L , et al. Novel risk loci for rheumatoid arthritis in Han Chinese and congruence with risk variants in Europeans. Arthritis Rheumatol. 2014;66:1121‐1132. 24782177 10.1002/art.38353

[27] Elgueta R , Marks E , Nowak E , et al. CCR6‐dependent positioning of memory B cells is essential for their ability to mount a recall response to antigen. J Immunol. 2015;194:505‐513. 25505290 10.4049/jimmunol.1401553 PMC4282958

[28] Manthey HD , Cochain C , Barnsteiner S , et al. CCR6 selectively promotes monocyte mediated inflammation and atherogenesis in mice. Thromb Haemost. 2013;110:1267‐1277. 24114205 10.1160/TH13-01-0017

[29] Singh TP , Zhang HH , Borek I , et al. Monocyte‐derived inflammatory Langerhans cells and dermal dendritic cells mediate psoriasis‐like inflammation. Nat Commun 2016;7:13581. 27982014 10.1038/ncomms13581 PMC5171657

[30] Wen H , Hogaboam CM , Lukacs NW , Cook DN , Lira SA , Kunkel SL . The chemokine receptor CCR6 is an important component of the innate immune response. Eur J Immunol. 2007;37:2487‐2498. 17694574 10.1002/eji.200737370

[31] Komatsu N , Mariotti‐Ferrandiz ME , Wang Y , Malissen B , Waldmann H , Hori S . Heterogeneity of natural Foxp3 + T cells: a committed regulatory T‐cell lineage and an uncommitted minor population retaining plasticity. Proc Natl Acad Sci U S A. 2009;106:1903‐1908. 19174509 10.1073/pnas.0811556106 PMC2644136

[32] Paulissen SMJ , van Hamburg JP , Davelaar N , et al. Th cell populations distinguish ACPA‐positive from ACPA negative rheumatoid arthritis. Arthritis Res Ther. 2015;17:344. 26617177 10.1186/s13075-015-0800-5 PMC4663738

